# Hybrid Inorganic Complexes as Cancer Therapeutic Agents: In-vitro Validation

**DOI:** 10.7150/ntno.81557

**Published:** 2023-03-11

**Authors:** Murlidhar A Betallu, Shaileshkumar R Bhalara, Kailash B Sapnar, Vijay B Tadke, Keerti Meena, Ananya Srivastava, Gopal C Kundu, Mahadeo Gorain

**Affiliations:** 1 Department of Chemistry, Fergusson College, Pune, 411004, Maharashtra, India; 2 Laboratory of Tumor Biology, Angiogenesis and Nanomedicine Research, National Centre for Cell Science, Pune, 411007, Maharashtra, India; 3 Department of Physics, Fergusson College, Pune, 411004, Maharashtra, India; 4 School of Biotechnology and Kalinga Institute of Medical Sciences (KIMS), KIIT Deemed to be University, Institute of Eminence, Bhubaneswar 751 024, India; 5 BP Lab, Jamdoli, Jaipur, Rajasthan 302031, India; 6 Department of Chemistry, Institute of Science, Banaras Hindu University, Varanasi, Uttar Pradesh, India

**Keywords:** Transition - magnesium tartarate complexes, bidentate tartarate ligand, octahedral tartarate complexes, anticancer activity.

## Abstract

A series of novel mixed transition metal-Magnesium tartarate complexes of general formulation [MMg(C_4_H_4_O_6_)_2_^.^xH_2_O] (where M = Mn, Fe, Co, Ni, Cu and Zn) is prepared with bidentate tartarate ligand. The synthesized complexes (C1 to C6) are characterized by various analytical techniques such as Elemental analysis, Thermo gravimetric analysis, FT-IR Spectroscopy, X-ray Diffraction, Magnetic susceptibility study etc. All complexes exhibit the composition MMgL_2_ where M = Mn(II), Fe(II), Co(II), Ni(II), Cu(II) and Zn(II) and L = bidentate tartarate ligand. Analytical data reveals all complexes possesses 1:1 (metal: ligand) ratio. FT-IR spectral study shows that bidentate tartarate ligand coordinate with metal ion in a bidentate manner through two oxygen atoms. Thermo gravimetric analysis of all complexes shows that degradation curves of complexes agrees with recommended formulae of the complexes. X-ray diffraction technique suggests that all complexes (C1 to C6) are polycrystalline in nature. All newly synthesized metal tartarate complexes and ligand were screened* in vitro* for their anticancer activity against human breast cancer (MDA-MB-231) cell line. The bioassays of all these complexes showed C3 (Co) and C5 (Cu) Mg-tartarate complexes contains maximum antiproliferative activity at 200 µg/ml concentration on MDA-MB-231 cells as compared to other complexes. MDA-MB-231 cells treated with C3 (Co) and C5 (Cu) Mg-tartarate complexes also showed inhibition in cell migration.

## Introduction

There has been growing interest in the study of different transition metal complexes by using different ligands. Also lot of work has been done on such a metal complexes in the past several years such as different transition metal complexes using different schiff base ligand of 'N' and 'O' donor having different applications such as microbial and catalytic activity are reported.^1^-^4^ A number of hydrazone derivatives possess interesting bioactivity towards anti-bacterial, antifungal,^5^ anti-convulsant,^6^ anti-inflammatory^7^ and anti-malarial^8^ but the metal complexes containing bidentate tartarate ligand of 'O' donor atoms are not well reported.

Literature survey reveals only few tartarate complexes containing single transition metal, such as cobalt tartarate comprises optoelectronic application^9^. The anticancer studies of a Schiff base; (E)-2((2-hydroxybenzylidene)amino-3-mercaptopropanoic acid (H_2_L) (obtained from 2-hydroxybenzaldehyde and L-cysteine) and its transition metal complexes have been reported.^10^ Tartarate complexes containing mixed metal like alkali-alkali metal tartarates, alkali-transition metal tartarates, metal-alkaline earth metal tartarates, covers different mechanical, technological applications and microbial activity. For instance, sodium-potassium tartarate is reported for its ferroelectric application,^11^ Potassium-chromium tartarate is used in medicine, antimony-barium tartarate in veterinary drugs,^12^ also useful in science and technology such as ferroelectric applications.^13^-^17^

To develop anticancer agents is wide in scope. Over last several decades interface between Oncology and Medicine may provide the most prolific roles in discovery of novel anticancer agents. Transition metals such as iron, manganese and copper etc. are involved in various biological processes, starting from ETC (Electron Transport Chain) cycle to enzymatic reactions to various structural roles and their involvements in proteins.^18^ Various metal holding compounds provides many benefits over conventional carbon holding compounds in the development of new medicines. These benefits are due to their capability to linking ligands in the 3D configuration, therefore permitting functionalization of these groups can be specify to defined molecular targets.^19^

Few tartarate complexes of mixed alkaline earth and transition metal are reported.^20^-^22^ Hence, to find some new biologically active metal complexes we have synthesized and characterised tartarate complexes of mixed transition and magnesium metal containing bidentate tartarate ligand which coordinate through oxygen of carboxyl group with metal ions such as Mg(II), Mn(II), Fe(II), Co(II), Ni(II), Cu(II) and Zn(II).^20^-^24^ The synthesis of complexes by co-precipitation technique using water as a solvent minimises cost and maximises yield.^20^-^24^ The biological activity of metal ions with the tartarate ligand improves as compared to the ligand. Biomedical application of mixed metal tartarate complexes such as antibacterial and antifungal activity 20-24 and transition metal complexes of different ligands 25-34 are also reported.

To the best of our knowledge present paper is the first reported of the synthesis, characterization and *in vitro* anticancer activity of mixed transition metal-magnesium tartarate complexes of the type [MMg(C_4_H_4_O_6_)_2_^.^xH_2_O] which contain Mg as a fixed alkaline earth metal ion along with the series of transition metal ions i.e. M = Mn(II), Fe(II), Co(II), Ni(II), Cu(II) and Zn(II) bonded together through bidentate tartarate ligand. Tartarate is having donor site of two carboxyl oxygen atoms. These complexes are characterized by various analytical techniques such as elemental analysis, thermo gravimetric analysis, FT-IR Spectroscopy, X-ray Diffraction, magnetic susceptibility study. The synthesized metal tartarate complexes and ligand were screened* in vitro* for their anticancer activity against human breast cancer cell line MDA-MB-231 and muse embryonic NIH/3T3 cells.

## Results and Discussion

**Characterization of complexes:** Complexes are powdered solids, coloured and quite steady towards air and moisture at room temperature. Their decomposition temperature is also quite high.

**Elemental analysis:** Elemental analysis is the qualitative detection and quantitative determination of chemical elements (atoms, ions) in a sample. To detect an element, one should fix an appearance of an analytical signal: the formation of precipitate or characteristic crystals, color change, an isolation of gaseous products, an appearance of a definite line in spectrum, luminescence, etc. The elemental analysis made in weight per cent of tartarate complexes for metals, C and H are very well matched with the calculated ones. The data is summarised in Table 1. Analytical data shows that metal chelates have 1:1:2 stoichiometry, hence the synthesized tartarate complexes can be formulated as [MMg(C_4_H_4_O_6_)_2_^.^xH_2_O] where M = Mn(II), Fe(II), Co(II), Ni(II), Cu(II) and Zn(II).

**Figure 1:**
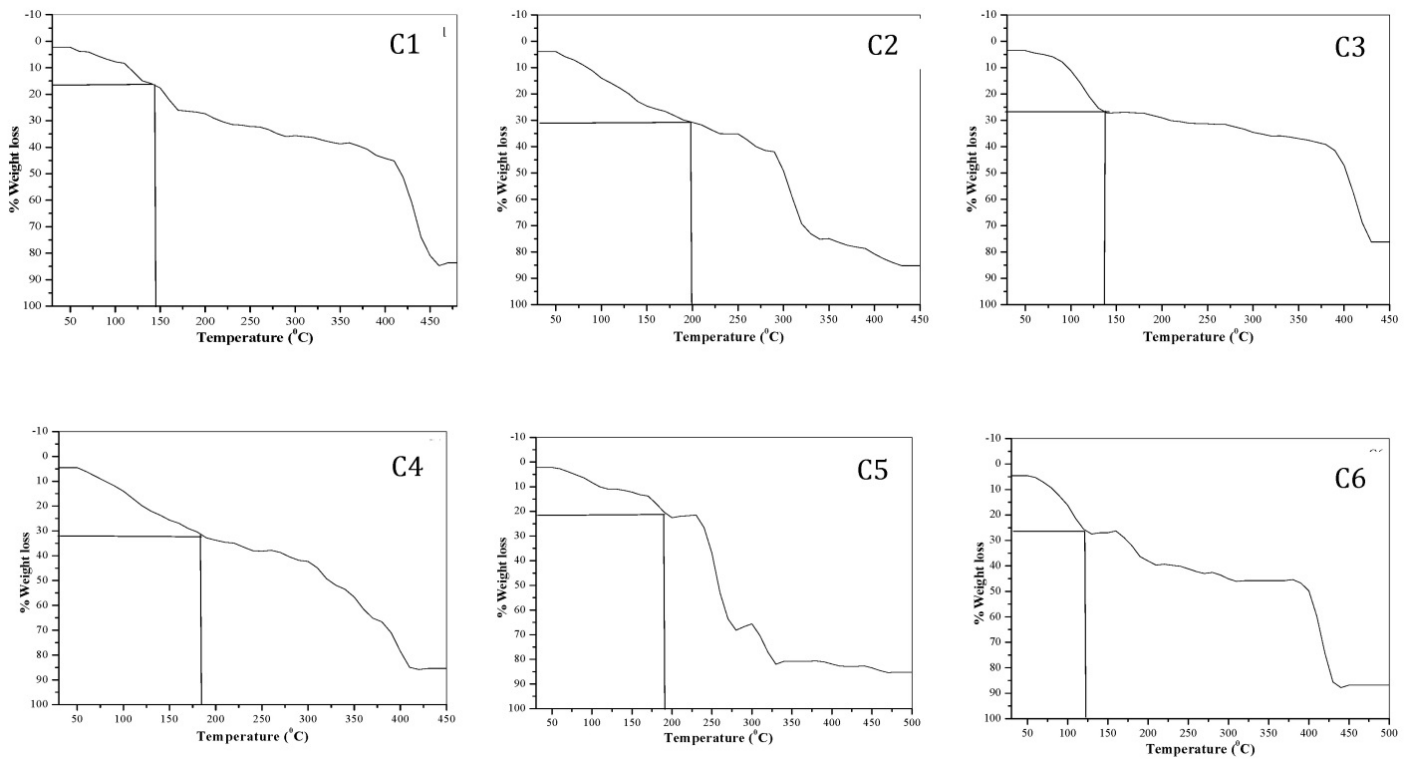
TGA curves of transition metal-Mg tartarate complexes (C1 to C6)

**Thermo gravimetric analysis:** Thermo gravimetric analysis data is summarized in Table 2. TGA curves of the complexes are shown in the Fig. 1. All the complexes have different number of water of hydration, hence it shows continuous loss of mass between temperature 60 to 150 ^o^C indicating the loss of water molecules from the tartarate complexes at this temperature.^20^-^24^, ^35^, ^36^ Water has many properties that are critical to maintaining life. It is a polar molecule, allowing for the formation of hydrogen bonds. Hydrogen bonds allow ions and other polar molecules to dissolve in water. Therefore, water is an excellent solvent.The % loss of water molecules for all complexes is well matched with the theoretical values. The oxidative decomposition of the ligand is observed between different temperature ranges of 150 to 250 ^o^C (Table 2).

**Figure 2:**
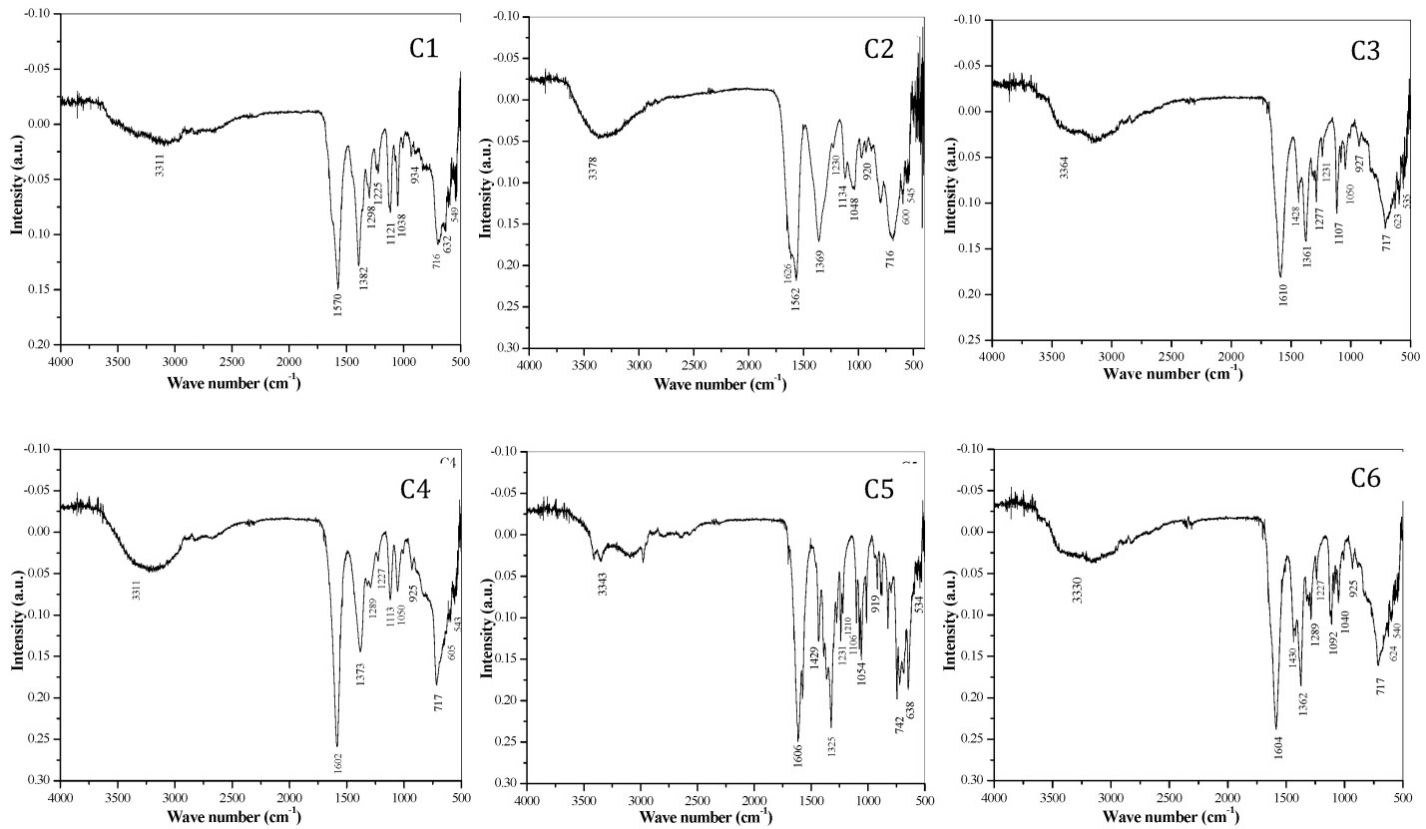
FT-IR spectra of transition metal-Mg tartarate complexes (C1 to C6) showing different stretching frequencies

**FT-IR spectral study:** FT-IR spectra of all complexes [Mn(II), Fe(II), Co(II), Ni(II), Cu(II) and Zn(II)] are shown in the Fig. 2. The bidentate linkage of metal ions at donor sites of the ligand tartrate was confirmed by IR spectral assessment of the ligand and its metal complexes. The observed absorption frequencies and their assignment in relation to their characteristic vibrational modes are given in Table 3. The strong intensity band at 1750 cm^-1^ in di- tartarate is shifted to lower wave numbers at ν sy (OCO) 1611 cm^-1^ and ν sy (OCO) 1440-1360 cm^-1^ for tartarate complexes which confirms the coordination of both carboxylate (COO-) groups in tartarate molecule and the metallic ions.

It was also reported that coordination of carboxylate (-COO-) group with metal results in lowering of both v (COO) frequencies due to transfer of electron density from carboxylate (-COO-) group to the metal ion in the complex. Later, on the based on difference between symmetric and asymmetric (C=O) stretching frequencies of tartarate complexes, bidentate nature of attachment of ligand to metal ion can be anticipated. The broad and strong trough positioned in between 3378-3311 cm^-1^ related to the hydroxyl (-OH) vibration frequency of water (H_2_O) and secondary alcoholic group (-CHOH) which shows that complexes are hydrous. The band at 549-534 cm^-1^ corresponds to the M-O bonding. The bands ranging from 1562-1038 cm^-1^ are the bands corresponding to the C-O bonding in the tartarate of the complexes.^9^, ^20^-^22^, ^24^, ^31^, ^34^-^38^

**Figure 3:**
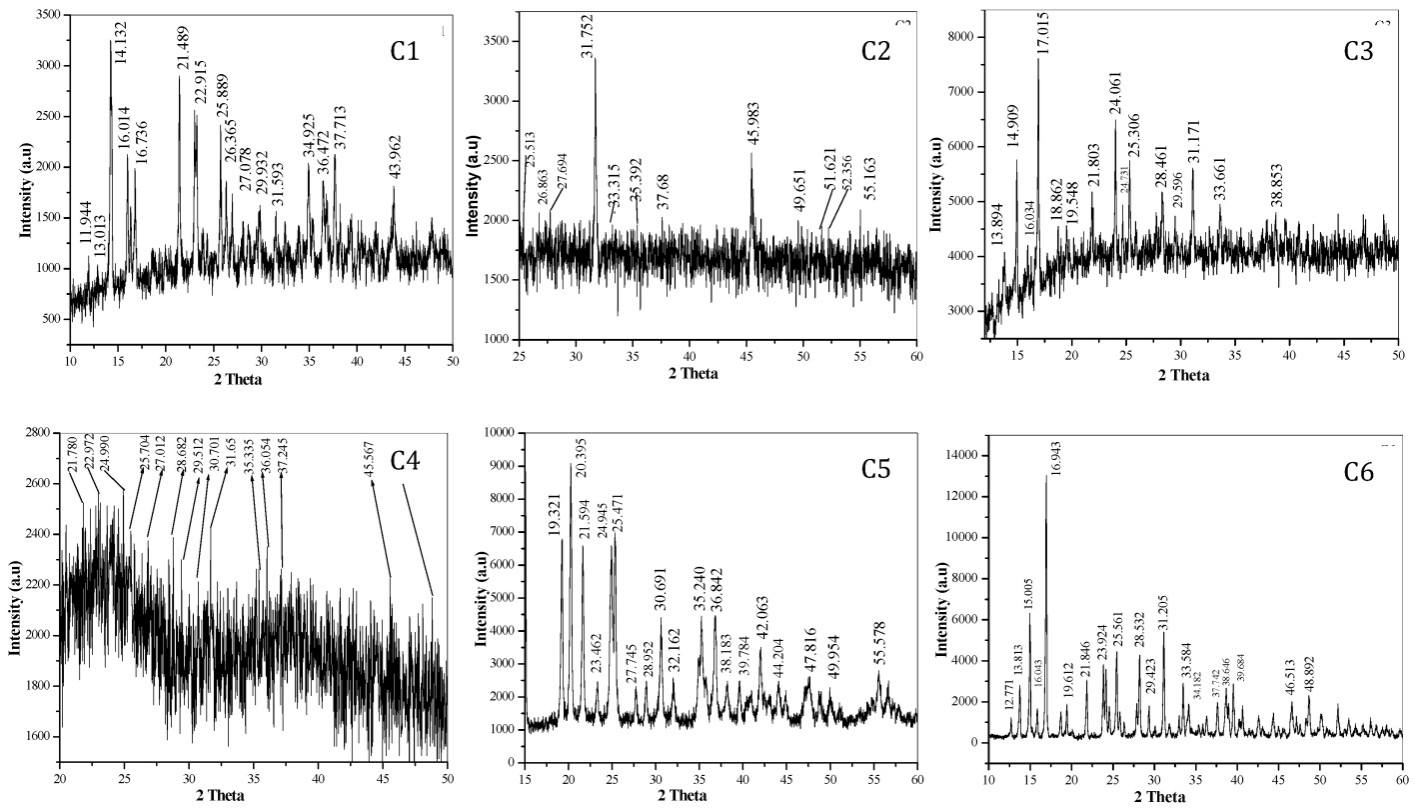
X-ray diffraction pattern showing 2θ value of transition metal-Mg tartarate complexes (C1 to C6)

**X-ray powder diffraction:** Nowadays, Powder XRD has gained attention as a technique to elucidate the structural data information of materials, especially in organic as well as inorganic compounds in the absence of single crystal XRD data. The X-ray powder diffraction pattern of these complexes are shown in fig. 3. The XRD pattern shows complexes are polycrystalline in nature. The observed'd' spacing values are given in Table 4, while particle size of all complexes (C1 to C6) calculated using formula (D = 0.89λ / βCosθ) are given in Table 5.^20^-^24^, ^35^, ^36^, ^37^, ^39^

**Magnetic susceptibility:** With respect to the number of unpaired electrons present in the complex (C1 to C6), their magnetic moment values are mentioned in Table 6. In case of C6, Zinc(II)-Mg(II) tartarate complex, the magnetic moment value is zero which reveals that no unpaired electrons are present in the complex and hence results diamagnetism.^20^-^22^, ^24^, ^35^, ^36^

**Figure 4:**
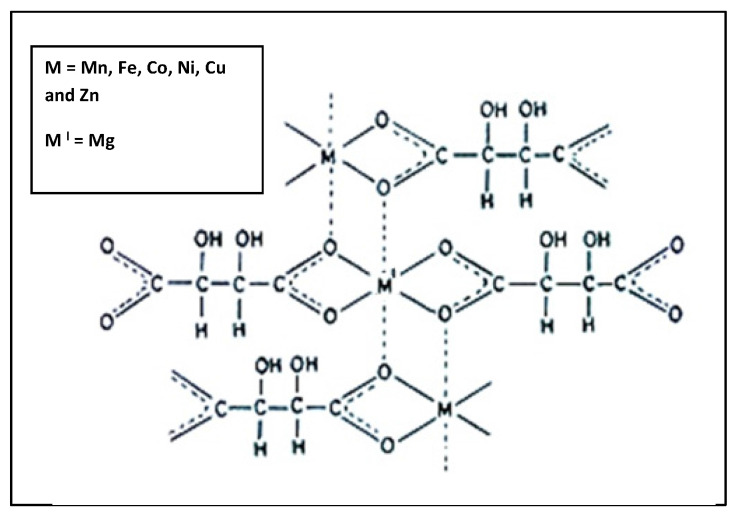
Probable polymeric octahedral structure of transition metal-Mg tartarate complexes (C1 to C6) showing attachment of ligand to the Metal(II) ion through two 'O' atom

**Structure of complex:** The IR spectral study suggest that the tartarate ligand coordinate with the metal ions in bidentate fashion through two oxygen donor sites, giving an uninterrupted polymeric structure having each metal ion surrounded by ligand in octahedral geometry (Fig. 4).^20^-^22^, ^24^

**Cell viability assay:** Metal-based drugs are increasing interest for pharmaceutical, inorganic and medicinal chemistry to development of novel drugs.^40^ However; major problems related to anti-cancer metallo drugs are their resistance, toxicity and other side-effects. Cellular redox homeostasis interference of cancer cells seems promising approach for cancer therapy because redox properties of metal containing compounds direct interacts and disturbs the cellular redox homeostasis which leads to cell death.^41^

**Figure 5:**
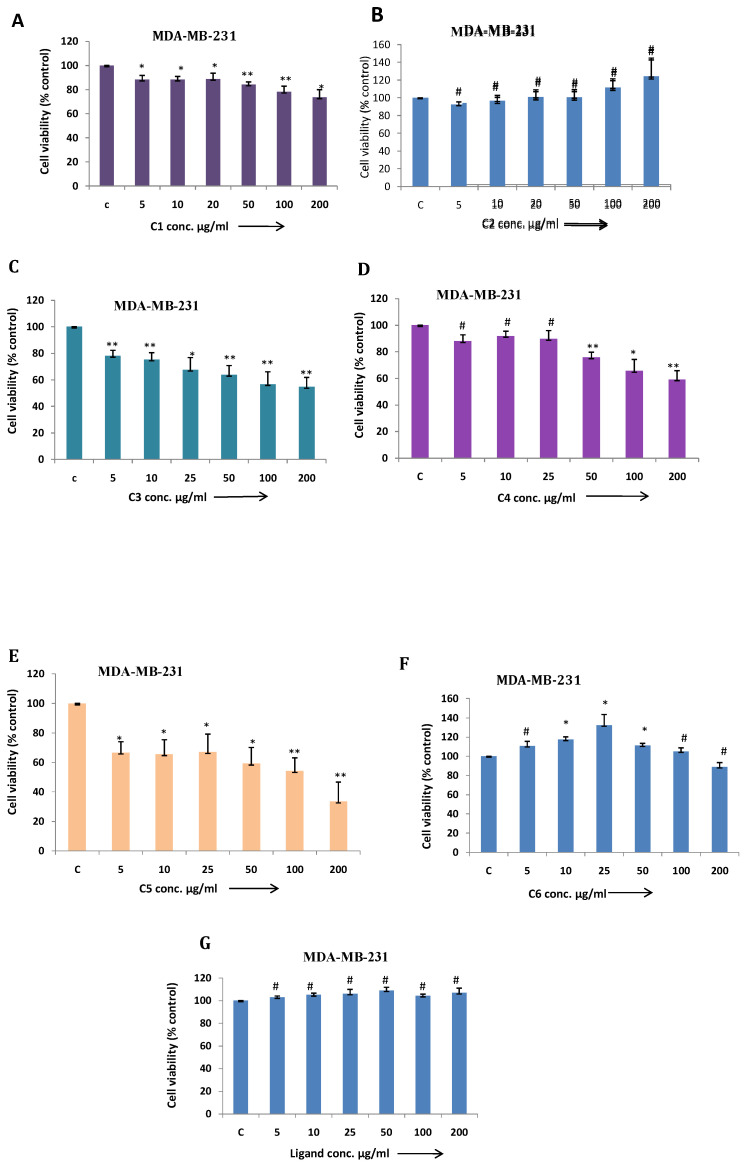
(a-g) MDA-MB-231 cells were treated with C1 to C6 complex compounds and ligand at indicated doses from 5 to 200 μg/ml for 24 h. and cell viability was determined by MTT assay. Bar graph denotes mean ± SEM (n=3). * *P* ≤ 0.05 vs. control. ** *P* ≤ 0.001 vs. control and #* P* >0.05 vs. control

**Figure 6:**
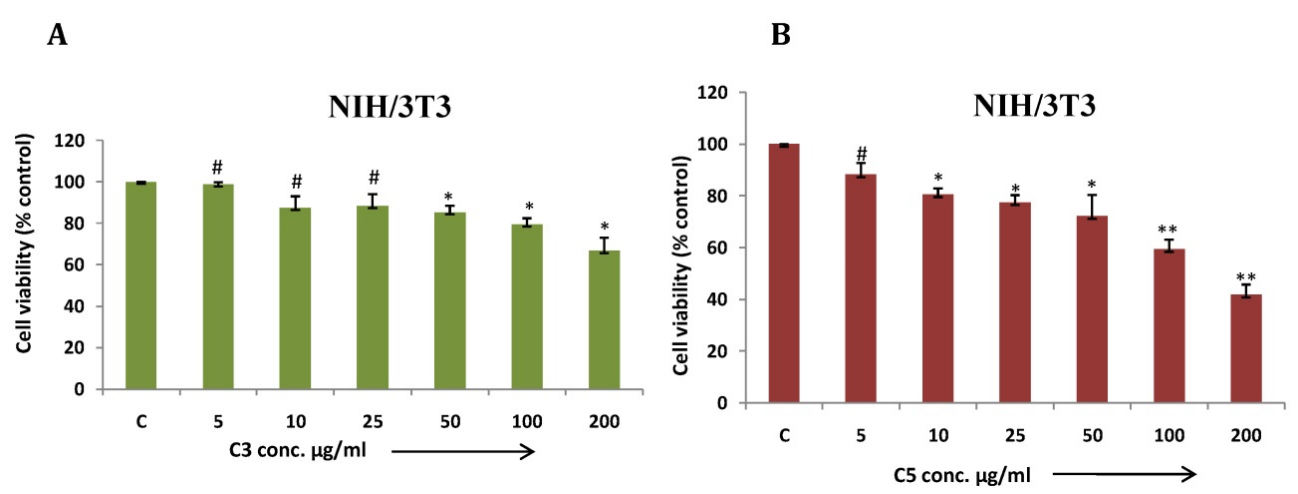
** (a & b)** NIH/3T3 cells were treated with C3 and C5 complex compounds and ligand at indicated doses from 5 to 200 μg/ml for 24 hrs and cell viability was determined by MTT assay. Bar graph denotes mean ± SEM (n=3). * *P* ≤ 0.05 vs. control. ** *P* ≤ 0.0001 vs. control and #* P* >0.05 vs. control

In this article, we demonstrated MDA-MB-231 cell viability treated with different metal based complexes C1 to C6 and ligand (Fig. 5 A-G) and as outcome C3 (Co) and C5 (Cu) Mg-tartarate complexes showed maximum antiproliferative activity at 200µg/ml (IC50 value >200 µg/ml and 150µg/ml C3 and C5 respectively). Anticancer activity of C5 (Cu)Mg-tartarate complex may be due to the presence of copper ion which promotes different mechanisms leading to production of reactive oxygen species (ROS) 42. Whereas mode of antiproliferative activity of C3 (Co)Mg-tartarate complex may due to two aspects: (i) Generation of ROS by catalytic auto oxidation process by schiff base complexes (ii) activation of Co(III) complexes in hypoxic environment by reduction to Co(II) and ligand release.^42^, ^43^ Other complexes showed less or no antiproliferative activity at the same concentrations as compared to C3 and C5 complexes. NIH/3T3 cell showed less toxic effect treated with C3 and C5 complexes as compared to MDA-MB-231 (Fig. 6A & 6B).

**Figure 7:**
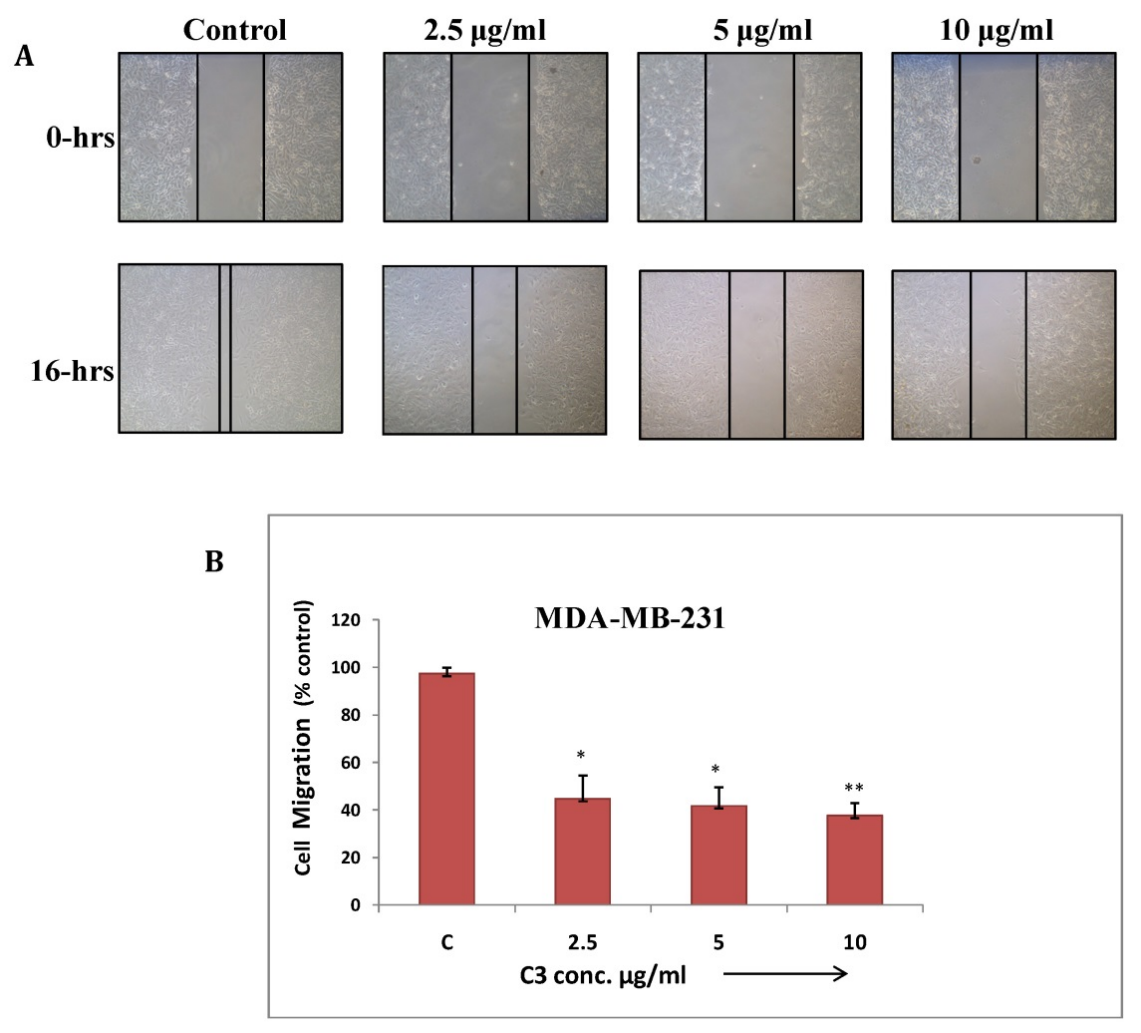
** (a)** Cell migration assay of MDA-MB-231 cells treated with C3 compound from 2.5 to 10µg/ml for 16 hrs. Migration of cells to the wounded area was observed by using phase contrast microscope (Nikon) and photographed (10x magnification) **(b)** Distance migrated was measured by image-Pro plus software. Quantitation of per cent cells migration where bar graph denotes mean ± SEM (n=3). * *P* ≤ 0.006 vs. control and ** *P* = 0.0005 vs. control

**Figure 8:**
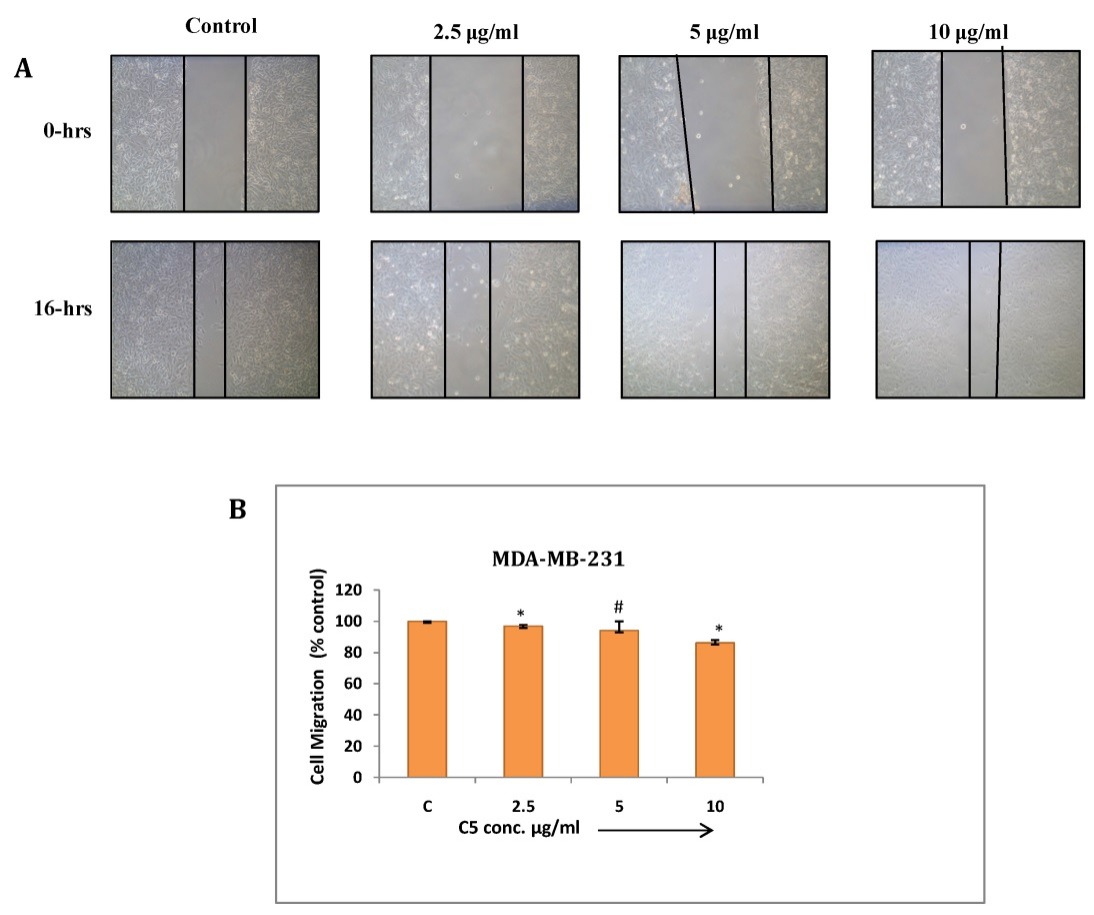
** (a)** Cell migration assay of MDA-MB-231 cells treated with C5 compound from 2.5 to 10µg/ml for 16 hrs. Migration of cells to the wounded area was observed by using phase contrast microscope (Nikon) and photographed (10x magnification) **(b)** Distance migrated was measured by image-Pro plus software. Quantitation of per cent cells migration where bar graph denotes mean ± SEM (n=3). # *P* > 0.05 vs. control and * *P* ≤ 0.002 vs. control.

**Wound healing assay:** Cancer cells have tendency to migrate from primary site and metastasize at different tissues and organs which makes it more aggressive and uncontrollable. This assay has been performed to study effect of C3 and C5 complexes on human breast cancer (MDA-MB-231) cell migration. The result showed significant inhibition in migration at maximum concentrations of C3 and C5 (7.5µg/ml) (Fig. 7 and 8). C3 (Co) Complex and C5 (Cu) magnesium complex showed decrease in migration may be due to production of ROS and other biochemical imbalance.

## Conclusion

In this report the successful preparation of mixed M(II)-Mg(II) coordinated complexes (where M = Mn, Fe, Co, Ni, Cu and Zn) by co-precipitation technique in the ratio of MMgL_2_. (where L=Ligand). We observed following results- the elemental analysis of all complexes and C, H data is very well matched with expected ones. Thermo gravimetric analysis gives the information about number of water molecules and oxidative decomposition of the ligand. The IR spectral study shows that the tartarate ligand coordinate with the metal ions in bidentate linkage through two oxygen donor sites, forming an uninterrupted polymeric structure in which each metal ion is surrounded by ligand in octahedral manner. The X-ray powder diffraction pattern reveals complexes are polycrystalline in nature. Over all study suggest that the metal chelate exhibited polymeric octahedral structure (Fig. 6). A magnetic moment value suggests the paramagnetism and diamagnetism of precursors. The FT-IR spectrum of the complexes has asymmetric broad trough positioned in between 3378-3311 cm^-1^ relates to O-H bonding confirms the hydrous nature of the tartarate complexes. The spectrum also shows the absorption at 549-534 cm^-1^ which reveals the presence of M-O bond. C2 have higher absorption in the UV region at about 386nm of the spectrum, which makes the material to be appropriate for devices for better absorption of UV radiation hence it can be used as an UV filters. The energy gap (Eg) of the complex C2 inferred as 3.21eV which shows that the complex C2 is semiconductor. This is the first report which demonstrates among six transition metals-magnesium tartarate complexes studied; C3 and C5 complexes significantly affect MDA-MB-231 cell viability and its migration.

## Experimental Section

**Synthesis of precursors:** All the complexes (C1 to C6) were prepared by a simple co-precipitation technique.

**Synthesis of complex C1:** The two metal salts aqueous solution i.e. MgCl_2_.6H_2_O (Sigma Aldrich) and MnCl_2_^.^4H_2_O (Sigma Aldrich) were mixed homogeneously in a fixed molar ratio. The pH of medium was adjusted to values (pH < 6) therefore precipitate of hydroxide not formed. The solution is then vigorously agitated with the help of magnetic stirrer in N_2_ atmosphere and the temperature of the solution was maintained at 60 ^o^C. The sodium tartarate (15%) solution is then slowly added to it with constant stirring till a permanent precipitate occurs. Acetone is added in equal amount to solution, where addition of acetone not only ensures a high yield, but also influences more homogeneous, stoichiometric, fine grained powders. The precipitate is filtered after stirring it more for 30 minutes. The filtrate is checked for Mg^2+^ and Mn^2+^ ions whose absence ensured complete co-precipitation. The precipitate is washed with acetone and then dried at room temperature, and then preserved in desiccators. All the tartarate metal complexes (C2 to C6) are prepared using similar general procedure.^20^-^22^, ^24^ All the synthesized metal complexes are obtained with good percentage yield. Details of complex composition are summarised in Table 7.

**Elemental analysis:** C, H analysis done on D:\CHNS2016\SP12042016r.mth of C. E. Instruments. The metal content present in these complexes were estimated using AAS instrument. Metal content of some complexes also have been done using gravimetric and volumetric analysis.

**Thermo gravimetric analysis:** The thermo gravimetric analytical measurements of complexes were obtained with Perkin-Elmer (Delta series-TGA7) instrument. All complexes are studied under static atmosphere of air using 30-50 mg samples and providing heating rate up to 10 degree / minute in the range of ambient to 500 ^o^C.

**FT-IR spectral study:** IR spectrum of precursors was taken by the instrument IR Affinity, Shimadzu make, Japan, Fourier transform infrared (FT-IR) spectral study was mainly used to predict different type of bonding in the complex from which the structure of the transition metal-magnesium tartarate complexes can be predicted. IR spectroscopic region used was in between 4000 cm^-1^ and 400 cm^-1^ on the spectrometer. The Infrared spectra and their bond assignment of fundamental frequencies are given in Table 3.

**X-ray powder diffraction:** X-ray powder diffraction (XRD) is a rapid analytical technique which is used primarily for phase identification of newly synthesized transition metal- magnesium tartarate complexes. The X-ray powder diffraction pattern of transition metal-magnesium tartarate complexes (C1 to C6) were recorded in ambient air at room temperature using RIGAKU ULTIMA IV instrument with Cu Kα radiation (λ = 1.5406 A^o^). The structural analysis of tartarate precursors was done by comparing the experimental d values and relative intensities with ASTM powder file. The d values were calculated using Bragg's equation, nλ = 2d sinθ, where d is the distance between the parallel diffraction planes, θ is the angle between the incident beam and the diffraction plane, λ is the wavelength of the X-ray used, n is the order of diffraction. Particle size of these composites was determined by using an expression D = 0.89 λ / (βcosθ) in A^o^.

Where, λ = wavelength (Cu Kα), β = Full width at the half- maximum of the high intensity peak in XRD pattern of tartarate complexes, θ = diffraction angle.

**Magnetic susceptibility:** The magnetic susceptibility measurements for transition metal-Mg tartarate have been done at 27 ±1 ^o^C on Faraday balance using 7000 gauss magnetic field.

**Cell viability assay:** Human breast cancer (MDA-MB-231) cell line and mouse embryonic (NIH/3T3) cell line obtained from American Type Culture Collection (ATCC, Manassas, VA, USA) and used for this assay.^44^,^45^ MDA-MB-231 cells were maintained in L15 medium contains 10% FBS, penicillin (100 U/ml) and streptomycin (100 mg/ml) under humidified atmosphere at 37 ^0^C. NIH/3T3 cells maintained in DMEM medium contains 10% FBS, penicillin (100 U/ml) and streptomycin (100 mg/ml) under humidified atmosphere at 37 ^0^C and 5% CO_2_. 1×10^4^ cells were (MDA-MB-231 and NIH/3T3) seeded per well in 96 well plate followed by 24 hours of incubation. Treatment of C1 to C6 and ligand were given at different concentrations from 5 to 200µg/ml. After 24 hours incubation, MTT (3-(4,5-dimethylthiazol-2-yl)-2,5-diphenyltetrazolium bromide) solution was added per well to achieve a final conc. of 0.5 mg/ml and incubated for 4 hours at 37 ^o^C.^44^-^47^ Isopropanol (solubilizing solution) was added to dissolve formazan crystals and absorbance was recorded at 570 nm on a micro plate reader (Epoch 2 Microplate Reader, BioTek, USA).

**Wound Healing Assay:** MDA-MB-231 cells were seeded in a 12 well plate and incubated till uniform monolayer achieved. Sterile T-200 micropipette tip was used to scrape wound in order to make wound of constant width. Cellular debris were removed by L15 basal medium followed by treatment of C3 and C5 (2.5 to 7.5 µg/ml) and incubated at 37^o^C for 16 hrs. Migration of cells to the wounded area was observed by using phase contrast microscope (Nikon) and photographed (10x magnification).^44^, ^45^, ^48^, ^49^ Distance migrated was measured by image-Pro plus software. Percentage of wound closure was estimated by: Wound closure Percentage = [1-(wound area at T_t_ / wound area at T_0_) × 100%, where, T_t_ is 16 hrs after wounding and T_0_ is the time immediately after wounding.

## Figures and Tables

**Table 1:** Elemental analysis data of transition metal-Mg tartarate complexes (C1 to C6)

Formula	Code	Mol. Wt.	C		H		Transition Metal (%)		Mg %	
			Obs.	Cal.	Obs.	Cal.	Obs.	Cal.	Obs.	Cal.
MnMg(C_4_H_4_O_6_)_2_^.^4H_2_O	**C1**	447	20.68	21.47	3.01	3.57	11.98	12.30	5.06	5.36
FeMg(C_4_H_4_O_6_)_2_^.^10H_2_O	**C2**	556	15.93	17.26	3.06	5.03	9.85	10.07	4.13	4.31
CoMg(C_4_H_4_O_6_)_2_^.^8H_2_O	**C3**	523	18.95	18.35	3.62	4.5	10.80	11.28	4.26	4.58
NiMg(C_4_H_4_O_6_)_2_^.^10H_2_O	**C4**	558.7	16.64	17.18	3.80	5.0	10.16	10.50	4.11	4.29
CuMg(C_4_H_4_O_6_)_2_^.^6H_2_O	**C5**	491.5	19.29	19.53	3.24	4.06	12.57	12.91	4.39	4.88
ZnMg(C_4_H_4_O_6_)_2_^.^8H_2_O	**C6**	529.4	17.76	18.13	3.35	4.53	12.12	12.35	4.18	4.53

**Table 2:** TGA data of transition metal-Mg tartarate complexes (C1 to C6) under static air

Complex	Sample	% mass loss	% mass loss	Temp. Range °C
		Obsd.	Calcd.	
MnMg(C_4_H_4_O_6_)_2_^.^4H2O	C1	16.55	16.10	5.-150
		38.50		150-300
		83.31		300-450
FeMg(C_4_H_4_O_6_)_2_^.^10H_2_O	C2	31.45	32.37	50-180
		74.00		180-330
		85.95		330-425
CoMg(C_4_H_4_O_6_)_2_^.^8H_2_O	C3	27.20	27.53	50-130
		36.30		130-300
		77.24		300-425
NiMg(C_4_H_4_O_6_)_2_^.^10H_2_O	C4	32.12	32.21	50-170
		53.37		170-330
		85.51		330-410
CuMg(C_4_H_4_O_6_)_2_^.^6H_2_O	C5	22.30	21.97	50-170
		67.38		170-280
		81.42		280-450
ZnMg(C_4_H_4_O_6_)_2_^.^8H_2_O	C6	26.82	27.20	50-120
		45.78		120-300
		87.10		300-450

**Table 3:** FT-IR spectral bands and their probable assignments in [cm^-1^] for transition Metal-Mg tartarate complexes (C1 to C6)

Sr. No	C1	C2	C3	C4	C5	C6	Assignments
1	3311	3378	3364	3311	3343	3330	ν asy (H-OH)
2	1570	1626	1610	1602	1606	1604	ν asy (C=O)
3		1562	1428		1429	1430	ν sy(C=O),asy(c-c)
4	1382	1369	1361	1373	1325	1362	ν sy c-o(carboxyl)
5	1298	1230	1277	1289	1231	1289	ν asy C-C
6	1225	1230	1231	1227	1210	1227	ν asy C-O
7	1121	1134	1107	1113	1106	1092	ν C-O(alcohol)
8	1038	1048	1050	1050	1054	1040	ν C-O(alcohol)
9	934	920	927	925	919	925	ν sym C-O),d(o-c=o)
10	716	716	717	717	742	717	ν sym C-C
11	632	600	623	605	638	624	ν (H-O-H)
12	549	545	535	543	534	540	ν (M-O) (C-C)

** C1**- MnMg(C_4_H_4_O_6_)_2_^.^4H_2_O, **C2**- FeMg(C_4_H_4_O_6_)_2_^.^10H_2_O, C**3**- CoMg(C_4_H_4_O_6_)_2_^.^8H_2_O, **C4**- NiMg(C_4_H_4_O_6_)_2_^.^10H_2_O, **C5**- CuMg(C_4_H_4_O_6_)_2_^.^6H_2_O, **C6**- ZnMg(C_4_H_4_O_6_)_2_^.^8H_2_O

**Table 4:** Observed 'd' spacing values of transition metal-Mg tartarate complexes (C1 to C6) in A^o^

C1	C2	C3	C4	C5	C6
7.4326	3.5032	6.3990	4.0770	4.6066	6.9576
6.8286	3.3283	5.9622	3.8844	4.3697	6.4363
6.2896	3.2316	5.5491	3.5745	4.1292	5.9233
5.5551	2.8268	5.2300	3.4765	3.8031	5.5412
5.3181	2.6980	4.7191	3.3119	3.5803	5.2495
4.1481	2.5440	4.5564	3.1213	3.5080	4.5417
3.8942	2.3939	4.0888	3.0361	3.2248	4.0813
3.4524	1.9997	3.7092	2.9202	3.0938	3.7306
3.3903	1.8415	3.6113	2.8304	2.9224	3.4945
3.3041	1.7755	3.5304	2.5482	2.7911	3.1384
2.9949	1.7528	3.1448	2.4991	2.5536	3.0445
2.8408	1.6697	3.0284	2.4212	2.4465	2.8746
2.5766		2.8784	1.9966	2.3635	2.6761
2.4712		2.6701	1.8685	2.2721	2.6305
2.3891		2.3251	1.8685	2.1541	2.3902
2.0654			1.7133	2.0547	2.3367
				1.9081	2.2778
				1.8313	1.9584
				1.6586	1.8685

**C1**- MnMg(C_4_H_4_O_6_)_2_^.^4H_2_O, **C2**- FeMg(C_4_H_4_O_6_)_2_^.^10H_2_O, **C3**- CoMg(C_4_H_4_O_6_)_2_^.^8H_2_O, **C4**- NiMg(C_4_H_4_O_6_)_2_^.^10H_2_O, **C5**- CuMg(C_4_H_4_O_6_)_2_^.^6H_2_O, **C6**- ZnMg(C_4_H_4_O_6_)_2_^.^8H_2_O

**Table 5:** Observed particle size of transition metal-Mg tartarate complexes (C1-C6) D = 0.89λ / (βcosθ)

Complex code	C1	C2	C3	C4	C5	C6
Particle size (A^o^)	605.51	169.01	141.10	152.40	757.16	982.44

**Table 6:** Magnetic moment of transition metal-Mg tartarate complexes (C1 to C6)

Complex code	C1	C2	C3	C4	C5	C6
Magnetic moment (µ) B.M	5.73	4.69	3.62	2.71	1.74	0.00

**Table 7:** Complex composition of transition metal-Mg tartarate complexes (C1 to C6)

Complex	Code	Colour	Mol. Wt.	Amount of MgCl_2_6H_2_O (gm)	Amount of metal salt (gm)	Tartarate solution added	% Yield of complex
MnMg(C_4_H_4_O_6_)_2_^.^4H_2_O	**C1**	Flesh White	447	6.822	MnCl_2_^.^4H_2_O 6.644	15%	74.55%
FeMg(C_4_H_4_O_6_)_2_^.^10H_2_O	**C2**	Yellow	556	5.484	FeCl_3_ 4.376	15%	65.83%
CoMg(C_4_H_4_O_6_)_2_^.^8H_2_O	**C3**	Pink	523	5.830	CoCl_2_^.^6H_2_O 6.823	15%	78.28%
NiMg(C_4_H_4_O_6_)_2_^.^10H_2_O	**C4**	Faint Green	558.7	5.458	NiCl_2_^.^6H_2_O 6.381	15%	70.58%
CuMg(C_4_H_4_O_6_)_2_^.^6H_2_O	**C5**	Blue	491.5	6.204	CuCl_2_^.^2H_2_O 5.202	15%	66.75%
ZnMg(C_4_H_4_O_6_)_2_^.^8H_2_O	**C6**	White	529.4	5.760	ZnCl_2_ 8.147	15%	73.52%

## Abbreviations

FT-IR: Fourier Transform-Infra red
TGA: Thermo Gravimetric Analysis
eV: electro Volt
Nm: Nanometre
